# Paracrine effect of GTP cyclohydrolase and angiopoietin-1 interaction in stromal fibroblasts on tumor Tie2 activation and breast cancer growth

**DOI:** 10.18632/oncotarget.6981

**Published:** 2016-01-22

**Authors:** Liye Chen, Xin Zeng, Esther Kleibeuker, Francesca Buffa, Alessandro Barberis, Russell D. Leek, Ioannis Roxanis, Wei Zhang, Andrew Worth, John S. Beech, Adrian L. Harris, Shijie Cai

**Affiliations:** ^1^ Molecular Oncology Laboratories, Department of Oncology, Weatherall Institute of Molecular Medicine, University of Oxford, John Radcliffe Hospital, Oxford OX3 9DS, UK; ^2^ Department of Cellular Pathology, Oxford University Hospitals and NIHR Biomedical Research Centre, John Radcliffe Hospital, Oxford OX3 9DU, UK; ^3^ Nuffield Department of Obstetrics and Gynaecology, John Radcliffe Hospital, Oxford OX3 9DU, UK; ^4^ Jenner Institute, Nuffield Department of Medicine, University of Oxford, Oxford OX3 7DQ, UK; ^5^ Gray Institute for Radiation Oncology and Biology, Department of Oncology, University of Oxford, Oxford OX3 7DQ, UK; ^6^ Current address: Xiamen Institute for Diabetes Research, The First Affiliated Hospital of Xiamen University, Xiamen, China

**Keywords:** cancer-associated fibroblasts, RTK Tie2, angiopoietin, GTP cyclohydrolase, breast cancer

## Abstract

Cancer-associated fibroblasts (CAFs) play a key role in promoting tumor growth, acting through complex paracrine regulation. GTP cyclohydrolase (GTPCH) expression for tetrahydrobiopterin synthesis in tumor stroma is implicated in angiogenesis and tumor development. However, the clinical significance of GTPCH expression in breast cancer is still elusive and how GTPCH regulates stromal fibroblast and tumor cell communication remains unknown. We found that GTPCH was upregulated in breast CAFs and epithelia, and high GTPCH RNA was significantly correlated with larger high grade tumors and worse prognosis. In cocultures, GTPCH expressing fibroblasts stimulated breast cancer cell proliferation and motility, cancer cell Tie2 phosphorylation and consequent downstream pathway activation. GTPCH interacted with Ang-1 in stromal fibroblasts and enhanced Ang-1 expression and function, which in turn phosphorylated tumor Tie2 and induced cell proliferation. In coimplantation xenografts, GTPCH in fibroblasts enhanced tumor growth, upregulating Ang-1 and alpha-smooth muscle actin mainly in fibroblast-like cells. GTPCH inhibition resulted in the attenuation of tumor growth and angiogenesis. GTPCH/Ang-1 interaction in stromal fibroblasts and activation of Tie2 on breast tumor cells could play an important role in supporting breast cancer growth. GTPCH may be an important mechanism of paracrine tumor growth and hence a target for therapy in breast cancer.

## INTRODUCTION

The development of carcinoma requires a complex surrounding microenvironment, which is composed of extracellular matrix (ECM), soluble growth factors, stromal cells and blood vessels [1]. Interaction between stromal cells and the tumor greatly influences tumor initiation, maintenance and progression [1].

Among tumor stromal cell types, fibroblasts are major components of the tumor mass. The activated cancer-associated fibroblasts (CAFs) are presented in the vicinity of the malignant lesion expressing myofibroblastic markers, often with alpha-smooth muscle actin (α-SMA). They exhibit enhanced proliferation and migratory phenotypes, supporting epithelial proliferation, malignant transformation, tumor vascularization and metastasis in prostate cancer [2], ovarian cancer [3] and breast cancer [4]. This is partly ascribing to dysregulation of their cellular signaling and their abnormal expression of angiogenic factors [5]. Clinical significance of CAFs has been established; they are correlated with poor prognosis in breast carcinoma [6, 7].

GTP cyclohydrolase (GTPCH) (*GCH; EC 3.5.4.16)* is the rate-limiting enzyme for *de novo* tetrahydrobiopterin (BH4) and neopterin synthesis [8]. GTPCH activity is tightly regulated under physiological conditions but greatly increased in cancer [9]. Our group has previously demonstrated that metabolic GTPCH expression in fibroblasts promotes tumor stroma growth partially by inducing angiogenesis [10]. These findings were confirmed recently by others [11]. However, GTPCH expression in breast cancer and the mechanisms by which GTPCH operates in the tumor microenvironment are largely unknown. In pilot screening studies we found that the medium from GTPCH-expressing fibroblasts induced phosphorylation of Tie2 in breast cancer cell lines and investigated the mechanism further.

Receptor tyrosine kinases (RTKs) play a key role in tumor development. Tie2, a transmembrane RTK, presents predominantly on vascular endothelial cells and is essential for the initiation of angiogenesis [12, 13]. Beyond the expression in the vascular system, Tie2 is detected in certain tumor cell types, such as brain [14], melanoma [15], ovarian [16] and breast cancer [17, 18].

There are three known human Tie2 ligands - angiopoietin-1 (Ang-1), Ang-2 and Ang-4 (the orthologue of murine Ang-3), involved in vessel development [19–21]. Ang-1 is expressed primarily in fibroblasts, pericytes, and smooth muscle cells, and maintains endothelial cell survival. It induces vessel sprouting, maintains perivascular mural cell coverage [19], and is recognized to play a role in stabilizing tumor vessel formation [22].

However, aberrant Ang-1 overexpression in tumor remains controversial. Ang-1-expressing breast cancer cells delay xenograft tumor growth due to increased pericyte recruitment in tumor vessels [23, 24], which benefits tumor perfusion and enhances the potency of anti-cancer chemotherapy in colorectal and prostate xenografts [25] or radiation therapy in a glioblastoma model [22]. In contrast, upregulation of Ang-1 accelerated mammary tumor growth and enlarged tumor vessel lumens [26], which may enable tumor cells to become more accessible to the adjacent blood stream for metastasis to a distant site [27]. In response to VEGF blockade in tumor intervention, tumor Ang-1/Tie2 compensated by inducing vessel remodeling and protecting the vasculature from regression [28]. These contradictory observations may be attributed to tumor types studied in different tumor microenvironments.

Considering fibroblast is the main source of Ang-1 and stromal fibroblast-expressing GTPCH induced angiogenesis in our previous work [10], we set out to (1) determine the location of GTPCH expression and its correlation with clinicopathology; (2) explore the paracrine effect of GTPCH and Ang-1 expression in stromal fibroblasts and mechanisms involving breast cancer growth, and (3) demonstrate GTPCH potential role as a therapy target.

## RESULTS

### Human GTPCH expression in stromal and epithelial cells in breast cancer

**Figure 1:**
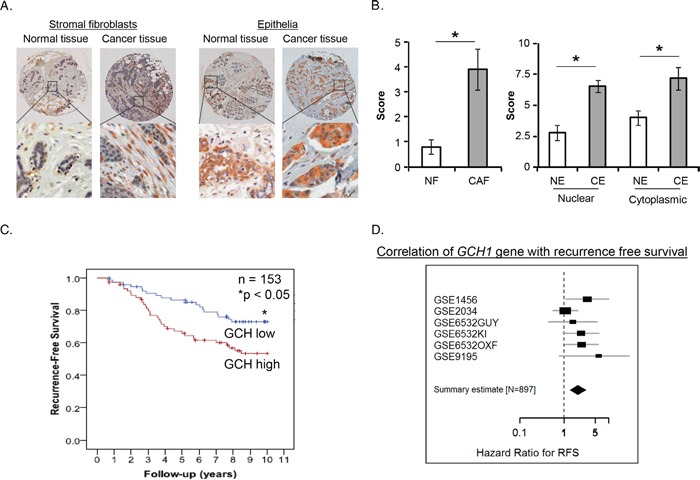
Prognostic significance of GTPCH expression in breast cancer patients **(A).** The breast cancer profiling TMA included carcinoma and normal tissue samples from individual patients (n = 21). GTPCH localized in various cellular compartments (brown color). **(B).** GTPCH presented predominantly in the fibroblasts and epithelia in breast cancer compared to normal tissue samples. All data are shown as mean ± SEM (*p <0.05 vs. NF or NE, n = 10). NF – normal fibroblasts; CF – cancer fibroblasts; NE – normal epithelium; CE – cancer epithelium. **(C).** High levels of *GCH1* gene expression are associated with worse survival in breast cancer patients. Kaplan-Meier curves showing 153 subjects with high or low expression of *GCH1* gene as determined by median expression value in breast cancer. *p* value was computed by a Log-rank test. **(D).** Recurrence-free survival in 897 breast cancer cases. A Forest plot showing meta-analysis performed on 6 curated retrospective breast cancer datasets deposited in the gene expression omnibus (http://www.ncbi.nlm.nih.gov/geo/) repository (provide IDs if taken out from figure GSE1456, GSE2034, GSE6532, GSE9195). Identifiers are shown for the datasets. Hazard-ratio (HR) for recurrence-free survival (good prognosis if HR < 1, worse prognosis if HR > 1), with 95% confidence interval, is shown for each datasets and for the summary effect on 897 samples.

We analyzed expression of GTPCH by immunochemistry in a set of 21 tissue microarrays (TMA) including normal breast and breast cancer. GTPCH was expressed in a variety of the cellular compartments of the tissues, including inflammatory cells, endothelia, stromal fibroblasts and epithelia (Figure 1A). A different pattern of the expression in stromal fibroblasts or epithelia between breast cancer and normal breast was prominently displayed, extensively distributed in the former and narrowly confined in the latter (Figure 1A). Scores for GTPCH expression in breast CAF and epithelia were twice those in the normal breast (Figure 1B), demonstrating that GTPCH upregulation in breast CAFs and epithelia is commonly present in breast cancer.

To investigate the relationship of human *GCH1* (the gene encoding GTPCH) expression with the prognosis of breast cancer patients, we analyzed expression in a series of 153 patients with complete follow-up and demographics as published previously [29]. High *GCH1* (reporter *204224_s_at*) significantly correlated with larger, high grade tumors (Supplementary Table S1) and low recurrence-free survival (Figure 1C). Multivariate analysis, taking into account of estrogen receptor (ER) status as a binary covariate with tumor grade, size, nodal status and menopause, showed a significant independent correlation between high *GCH1* and worse survival rate (Supplementary Table S2). We validated this further by analyzing a series of 897 patients from several none-overlapping datasets [30] and confirmed a significant correlation of high *GCH1* with low recurrence-free survival Figure 1D). Furthermore, analysis of several sets of other gene arrays in over 3,000 breast cancer from Oncomine (web site https://www.oncomine.org/. ©2008-11 Compendia Bioscience, Inc.) showed that high *GCH1* significantly correlated with invasive high-grade tumors (Supplementary Table S3). The *GCH1* gene was also highly expressed in patients with ER or progesterone receptor (PR)-negative tumor status (Supplementary Table S3).

To study the link between GCH1 and Ang-1 in a tumor stromal compartment-specific manner, we analyzed their transcript levels in a public data set of human gene expression arrays from breast cancer stroma (Accession numbers: GSE9014). Essentially, there was a statistically significant correlation between *GCH1* and *Ang-1* expression, emphasizing the potential clinical relevance of a *GCH1/Ang-1* connection in tumor stroma (Supplementary Figure S1A). Furthermore, high *GCH1* in tumor stroma was significantly correlated with the phenotype of ER-breast cancer (Supplementary Figure S1B). However, the *Ang-1* gene alone was not expressed differentially within the tumor subtypes (Supplementary Figure S1C-E).

### Paracrine effects of GTPCH expression in stromal fibroblasts on tumor cell proliferation and migration in a coculture system

**Figure 2:**
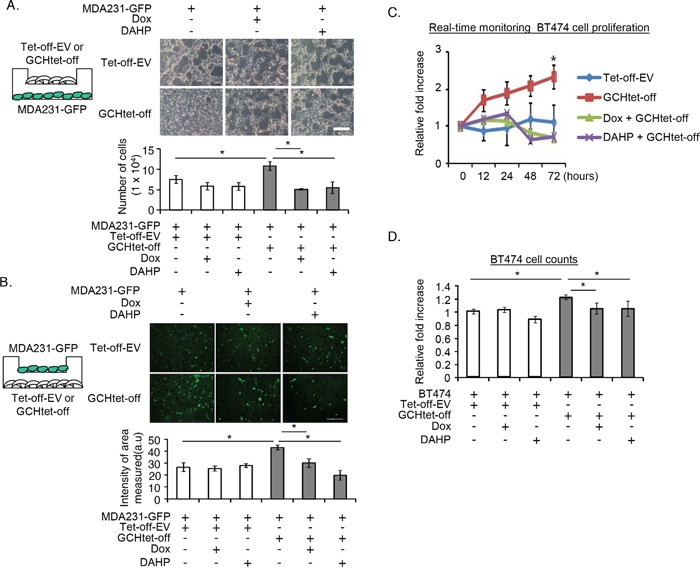
GTPCH-expressing fibroblasts promote breast cancer cell proliferation and migration **(A).** GCHtet-off or Tet-off-EV control fibroblasts were cultured in cell inserts (0.4 μm pore size) for 48 hours, followed by plating MDA231-GFP at the bottom of the well. Cells were incubated with serum-free medium ± DMSO vehicle control, Dox (1μg/ml), or DAHP (5 mM) for further 48 hours. Scale bar = 100 μM. **(B).** GCHtet-off or Tet-off-EV control fibroblasts were cultured at the bottom of wells for 48 hours, followed by placing MDA231-GFP in cell inserts (8 μm pore size). Cells were treated as for (A) for further 24 hours. **(C).** BT474 were cultured overnight and incubated with the medium of GCHtet-off or Tet-off-EV control fibroblasts ± DMSO vehicle control, Dox (1μg/ml), or DAHP (5 mM). Images were taken and analyzed by real-time Incucyte for 72 hours (C). BT474 were incubated with the medium of GCHtet-off or Tet-off-EV control fibroblasts after treated as for (C-a) for 48 hours, and the number of the cells were counted **(D).** All data are shown as mean ± SEM (*p <0.05 vs. DMSO control, Dox or DAHP, n = 6).

In order to rule out any compounding influence by other extracellular factors from the fibroblasts, we genetically modified NIH3T3-derived murine fibroblasts constitutively expressing GTPCH [10] to determine the role of the GTPCH/BH4 pathway on tumor development. The cells were chosen for this study because they can support breast and prostate tumor in coimplantation xenografts [31, 32]. Initially, we cultured GTPCH-expressing fibroblasts (GCHtet-off) or the Tet-off-EV control with MDA231-GFP, a GFP-transfected triple receptor negative breast epithelial cancer cell line [33], in transwell plates. The two cell types were separated by a porous membrane, which permitted diffusion of growth factors released from the fibroblasts to breast cancer cells. GTPCH-expressing fibroblasts cocultures significantly increased breast cancer cell proliferation by 20% and cell migration by 30% (Figure 2A and B). Switching off GTPCH by Dox or inhibiting its enzymatic activity for BH4 synthesis by DAHP (a GTPCH inhibitor) (Supplementary Table S2) suppressed these responses (Figure 2A and B).

We validated the protumor effects of GTPCH expression in stromal fibroblasts on cell proliferation of another breast cancer cell line – BT474, and monitored the cells using real-time Incucyte or counting them after the culture. Indeed, incubation with GTPCH-expressing fibroblast medium significantly increased BT474 cell numbers, compared to the Tet-off-EV control, Dox or DAHP treated medium (Figure 2C and D).

These findings indicate that expression of GTPCH in stromal fibroblasts supports cancer cell line growth *in vitro*.

### GTPCH expression and BH4 synthesis in stromal fibroblasts activates the Tie2 signaling in breast cancer cell lines

GTPCH-expressing fibroblasts, but not the control cells, synthesized BH4 (Supplementary Table S4) and triggered phosphorylation in cocultured tumor cell lines, MDA-MB231 and BT474, on Akt (Ser473) and ERK (Tyr202/Tyr204) (Supplementary Figure S2A). Conversely, pretreatment of the cancer cell lines with a PI3K inhibitor (GDC0941) or a MEK inhibitor (PD98059) respectively, decreased the phosphorylation to levels observed in the control (Supplementary Figure S2A), indicating the influence of the GTPCH/BH4 metabolism in stromal fibroblasts on the adjacent breast cancer cell PI3K and MAPK pathways.

We analyzed upstream signaling in breast cancer MDA-MB231 cells, with GTPCH-expressing fibroblasts ± Dox, DAHP or Ftase inhibitor III (a Ras inhibitor). GTPCH expression increased GTP-bound Ras in tumor cells (Supplementary Figure S2B), whereas switching off the expression by Dox or inhibiting BH4 synthesis by DAHP decreased GTP-bound Ras to the levels observed in the controls of Tet-off-EV and Ftase inhibitor III.

We used an anti-phospho-tyrosine receptor antibody array [for 42 human RTKs] to determine those RTKs activated in breast cancer cells by extracellular factors induced by GTPCH. We identified Tie2 on MDA-MB231 was the most prominent of the differentially phosphorylated RTKs, whereas the extent of phosphorylation to EphA7 was too weak to be recognized as a mechanism involving tumor signaling activation *in vitro* (Suppementary Figure S2C). The present study therefore focused on the tumor RTK Tie2 signaling.

**Figure 3:**
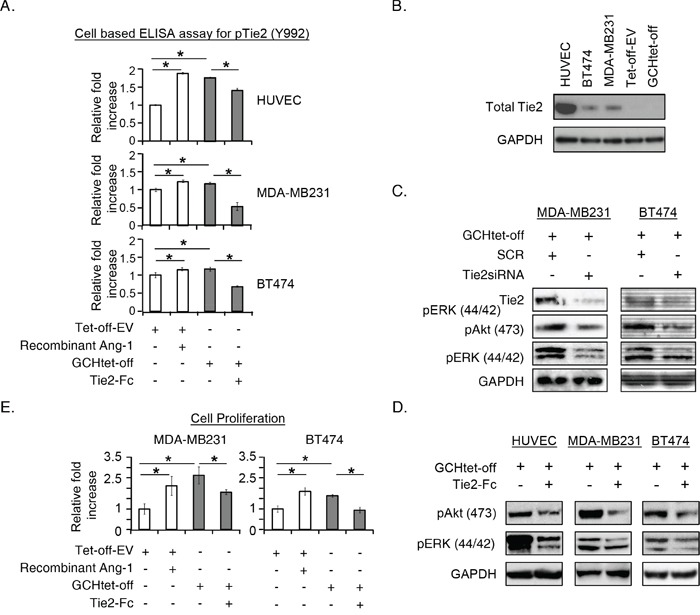
GTPCH-expressing fibroblasts induce Tie2 phosphorylation and activate Akt/ERK downstream pathways, in association with increased breast cancer cell proliferation **(A).** 1 × 10^4^ of MDA-MB231 or BT474 cells/well including control HUVEC were seeded in a 96-well plate. After incubation of these cells with the medium of GCHtet-off or Tet-off-EV control ± BSA vehicle control, recombinant Ang-1 (500 ng/ml) and Tie2-Fc (100ng/ml) for 20 min, cells were fixed and incubated with HRP-pTie2 (Y992) and AP-total Tie2. Fluorogenic signal was determined by normalizing fluorescence of pTie2 protein to total Tie2 after subtraction of background. **(B).** HUVEC, MDA-MB231, BT474, Tet-off-EV or GCHtet-off cells were cultured for 48 hours. Cell lysates were prepared for immunoblotting analysis with antibodies to total Tie2 and GAPDH, respectively (representative 3 independent experiments). **(C).** MDA-MB231 or BT474 were transfected with 10nM of *Tie2 siRNA* or *SCR siRNA* for 48 hours, or incubated with the medium of GCHtet-off ± BSA vehicle control or Tie2-Fc for 20 min. Cell lysates were prepared for immunoblotting analysis with antibodies to total Tie2, phospho-Akt/ERK and GAPDH, respectively (representative 2-3 independent experiments). **(D).** 1 × 10^5^ of MDA-MB231 or BT474 cells/well was seeded in 6-well plates after treated as for Figure 2C for 48 hours, the number of the cells were counted. All data are shown as mean ± SEM (*p <0.05 vs. BSA control, recombinant Ang-1 or Tie2-Fc, n = 3). **(E).** MDA-MB231 or BT474 were cultured with LS110 conditioned medium of GCH1siRNA or SCRsiRNA control for 48 hours, and numbers of the cells were counted (D). All data are shown as mean ± SEM (*p <0.05 vs. SCR control, GCHsiRNA n = 3).

We evaluated Tie2 activation with a cell-based ELISA. Essentially, the magnitude of the phosphorylation by GTPCH-expressing fibroblast medium on MDA231-GFP, BT474 and the control HUVEC was comparable to the recombinant Ang-1 stimulation (Figure 3A). Immunoblotting analysis confirmed the presence of endogenous Tie2 in MDA231-GFP and BT474, but absence in the fibroblasts (Figure 3B). Tie2 signaling is associated with the downstream cascade since knockdown with *Tie2siRNA* or blockage with the Tie2 recombinant antibody led to decreased phospho-Akt (Ser473) and ERK (Tyr202/Tyr204) to the levels observed in the controls of MDA231-GFP and BT474 cells (Figure 3C and D). Moreover, disruption of Tie2 signaling by a blocking recombinant antibody specific to Tie2 receptor significantly impaired GTPCH/BH4 induced breast cancer cell proliferation (Figure 3E).

Our results show the expression of GTPCH in stromal fibroblasts induced Tie2 phosphorylation and hence proliferation by a diffusible factor in breast cancer cell lines.

### Effect of endogenous GTPCH expression in human breast tissue-derived fibroblasts on breast cancer cell proliferation

**Figure 4:**
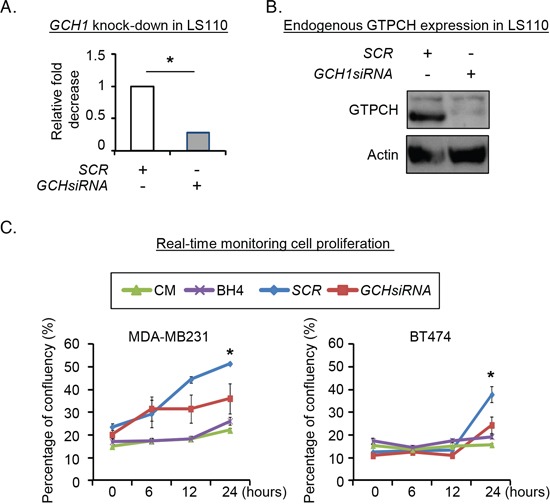
Evaluation of endogenous GTPCH expression in human breast tissue-derived fibroblasts on breast cancer cell proliferation Human breast fibroblast LS110 was transfected with *GCH siRNA* or *SCR siRNA, respectively* for 48hours. RNA from the cells were extracted and quantified for GCH1 mRNA **(A).** Cell lysates were prepared for immunoblotting analysis with antibodies to GTPCH or GAPDH (representative 3 independent experiments). **(B).** 2 × 10^4^ of MDA-MB231 or BT474 cells/well were seeded in 12-well plates and treated with conditioned media (CM) of *GCHsiRNA* or *SCRsiRNA* transfectants, or CM ± BH4 (100μM). Images were taken and analyzed by real-time Incucyte for 24 hours **(C).** All data are shown as mean ± SEM (*p <0.05 vs. SCR control, n = 3).

To validate the effect of endogenous fibroblast GTPCH on cell proliferation, we knocked down the gene in a human breast tissue-derived fibroblast cell line (Figure 4A and B) and cultured breast cancer cells with the conditioned medium (CM) for 24 hours, and monitored them in a real-time system. MDA-MB231 and BT474 cell proliferation in *GCHsiRNA* knockdown CM was reduced by approximately 20% compared to the *SCRsiRNA* control CM (Figure 4C). Thus GTPCH in fibroblasts induces tumor cell line growth. However, direct BH4 supplementation had a little impact on the cell expansion (Figure 4C). This may be because of rapid oxidation of extracellular BH4, compared to steady state intracellular synthesis with cells.

### GTPCH-associated Ang-1 secretion from stromal fibroblasts and Tie2 phosphorylation on tumor cells

**Figure 5:**
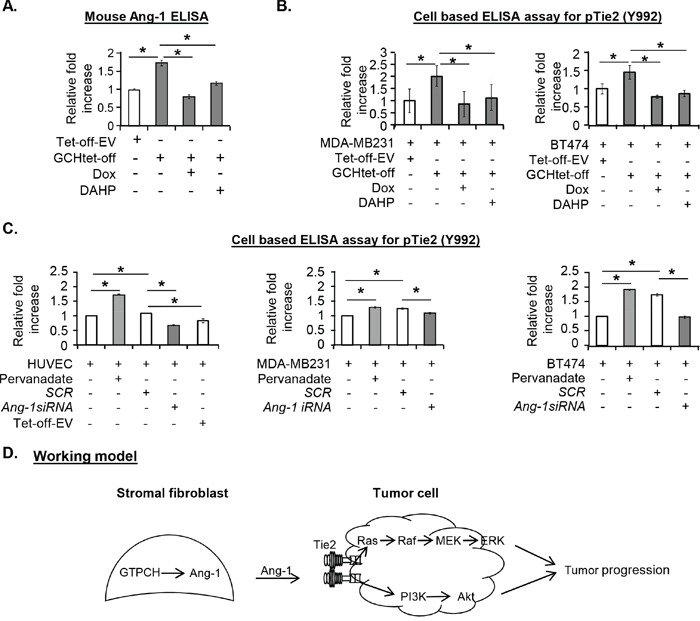
Biological effect of GTPCH-induced Ang-1 secretion on tumor Tie2 phosphorylation **(A).** Cell cultures were done and treated as for Figure 4. The culture medium was concentrated and measured for Ang-1 by ELISA. **(B).** 1 × 10^4^ of MDA-MB231 or BT474 cells/well was seeded in a 96-well plate. After incubation with the medium of the GCHtet-off or Tet-off-EV ± DMSO vehicle control, Dox (1μg/ml), or DAHP (5 mM) for 20 min, cells were fixed and treated as for Fig. 3. pTie2 was quantified using cell based ELISA assay. **(C).** Cell monolayers of HUVEC, MDA-MB231 or BT474 were incubated with the medium of *Ang-1siRNA* knockdown or SCR control in GCHtet-off or pervanadate positive control. After 20 min stimulation, pTie2 was quantified using cell based ELISA assay. All data are shown as mean ± SEM (*p <0.05 vs. DMSO control, Dox or DAHP, or siRNA knockdown vs SCRsiRNA, n = 3). **(D).** Schematic representation of a working model of the molecular mechanism by which formation of GTPCH/Ang-1 complex promotes tumour development.

Since Ang-1 binding is an initial step modulating the Tie2 signal, we measured levels of Ang-1 in culture medium using ELISA and showed that it was at least 50% greater in GTPCH-expressing fibroblasts than the control fibroblasts (Figure 5A). Conversely, Ang-1 was decreased in the Dox or DAHP-treated medium to levels that were observed in the control, confirming a close link between GTPCH and Ang-1 expression (Figure 5A).

To assess the impacts of stromal fibroblast-derived culture medium on tumor Tie2 activation in the cell line, we used a cell-based ELISA and found a comparable strength of Tie2 phosphorylation (Tyr992) by Ang-1 and pervanadate in HUVEC (Supplementary Figure S3A). GTPCH-expressing fibroblast CM activated Tie2 on HUVEC, MDA-MB231 and BT474 (Supplementary Figure S3A and Figure 5B). The phosphorylation was increased by 2-fold for MDA-MB231 and 1.5-fold for BT474, respectively, relatively to the control CM (Figure 5B). Treatment of the fibroblasts by Dox or DAHP reduced the effects of CM to baseline levels and shows that the phosphorylation is induced by GTPCH (Figure 5B).

The effect of CM on Tie2 induction on MDA-MB231, BT474 and HUVEC was mediated by Ang-1 because Ang-1 knockdown (Supplementary Figure S3B and S3C) in the fibroblasts led to a remarkable reduction of Tie2 phosphorylation by CM to levels observed in untreated cancer cells (Figure 5C).

Therefore, interplay between GTPCH and Ang-1 in stromal fibroblasts in conjunction with Tie2 activation on breast cancer cells potentially reveal a novel mechanism in facilitating the communication between stromal fibroblast and breast cancer cell *in vitro* (Figure 5D).

### GTPCH expression and BH4 synthesis in stromal fibroblasts promotes tumor growth and angiogenesis *in vivo*

**Figure 6:**
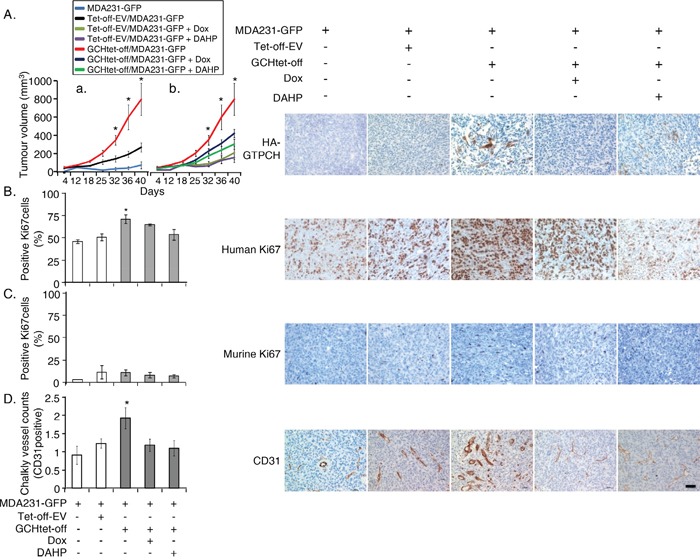
GTPCH-expressing fibroblasts accelerate breast cancer development and increase angiogenesis in mouse xenografts 1 × 10^6^ of MDA231-GFP alone, or mixed with 2 × 10^5^ of GCHtet-off cells or Tet-off-EV control at the ratio of 5:1 were respectively injected subcutaneously into flanks of 6-8 week old female Balb/c SCID mice (n = 5). 2 g/L of Dox or DAHP, or DMSO vehicle control was added to drinking water with 5% sucrose. Tumour volumes were monitored 2-3 times per week; when they reached the maximum permitted volumes (A), mice were sacrificed, and tumours were sectioned and stained immunohistochemically for HA-GTPCH (A), human Ki67 (B), murine Ki67 (C) and CD31 (D). Data are shown as the mean of 5 sections per animal ± SEM (*p <0.05 vs. DMSO control, Dox or DAHP treated tumoursHP, n = 5). Scale bar = 50 μM.

We implanted Balb/c SCID (5 mice in each group) with MDA231-GFP ± GCHtet-off or Tet-off-EV control fibroblasts at the ratio of 5:1 subcutaneously rather than orthotopically. A similar coimplant in the same number of mice per group was repeated. This approach avoids any confounding influence of the specific breast microenvironment and maximizes the opportunity to detect interaction between the transfected stromal fibroblasts and cancer cells. The tumor coimplants with GTPCH-expressing fibroblasts increased BH4 synthesis in the mass (Supplementary Table S5). All implants grew and they appeared by day 12 and expanded rapidly to the permitted maximum at day 40 (Figure 6A).

In those without fibroblasts not all animals (8/10) grew and they formed small MDA231-GFP tumors, but in coimplants of the Tet-off-EV tumors were larger (Figure 6Aa), demonstrating the protumorigenic effect of even GTPCH stromal fibroblasts on tumor growth. However, the tumor volumes were only 30% of that measured in the coimplants of GTPCH-expressing fibroblasts (Figure 6Aa). Therefore, although other factors are clearly present from control fibroblasts, when GTPCH is expressed the effects are much greater.

IHC staining showed that human Ki67 (a proliferation marker) in tumor GTPCH-expressing fibroblast coimplants increased by 25% compared to the MDA231-GFP alone or the control coimplants (Figure 6B), noting that murine Ki67 contributed less than 5% to each tumor mass (Figure 6C). The breast cancer cells represent the major cellular components of the tumor mass rather than the murine fibroblasts. CD31 (an angiogenesis marker) counts in the GTPCH-expressing coimplants were 1.5-fold greater than in the controls, and presented with an enlarged vessel lumen phenotype (Figure 6D).

**Figure 7:**
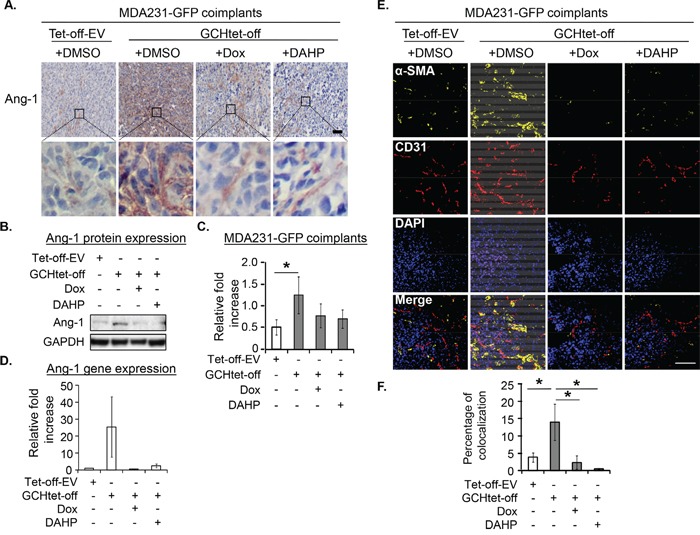
GTPCH expressing fibroblasts increases Ang-1 expression and vessel normalization in breast cancer mouse xenografts Xenografts were grown and treated as for Figure 5. Tumors were fixed, sectioned and stained immunohistochemically for Ang-1. Images are shown the representative section per animal **(A).** Tumor tissue lysates were prepared for SDS-PAGE and immunoblotted with antibodies to Ang-1 and GAPDH **(B).** Bands of intensity of Ang-1 were quantified on ImageJ software and normalized to GAPDH **(C).** RNA from tumor tissues was extracted and quantified for Ang-1 mRNA **(D).** The frozen sections were fixed and immunostained with antibodies to α-SMA (yellow), CD31 (red) and DAPI for DNA. Scale bar = 100 μM **(E).** Yellow and red overlapped signal was quantified by confocal computing programme **(F).** Data are shown as the mean of 5 animals per group ± SEM (*p <0.05 vs. DMSO control, Dox treated tumors).

After IHC staining, Ang-1 upregulation in the GTPCH-expressing fibroblast coimplants was mainly visualized in fibroblast-like cells (Figure 7A). Immunoblotting analysis confirmed a 20% increase of Ang-1 in the tumors compared to the control (Figure 7B and 7C). This change pattern echoed a degree of their Ang-1 mRNA expression although not significantly different due to intertumoral variation in each group (Figure 7D).

Whereas those animals fed with Dox or DAHP in drinking water to suppress the GTPCH/BH4 pathway had basal levels of BH4 (Supplementary Table S5) and halved the tumor growth, the effect on the control tumors growth was not significant (Figure 6Ab). IHC analysis confirmed downregulation of HA-GTPCH expression by Dox (Figure 6A), corresponding to a 10% reduction of human Ki67 (Figure 6B). Similarly, suppression by Dox or DAHP led to a 40% decrease in CD31 positive microvessel count (Figure 6D). Ang-1 showed a downward trend in both protein and gene expressions, although the difference did not reach statistical significance (Figure 7B-C).

We confirmed *in vitro* findings that Akt and ERK phosphorylation were significantly at least 2-fold and 1.5-fold greater, respectively, in the tumor coimplants of GTPCH-expressing fibroblasts than the controls (Supplementary Figure S4A-C). Although the phosphorylation in GTPCH-expressing coimplants decreased after the Dox or DAHP treatment, the statistical analysis was not significantly different between these groups (Supplementary Figure S4B and C).

### GTPCH expression drives tumor myofibroblast differentiation *in vivo*

**Figure 8:**
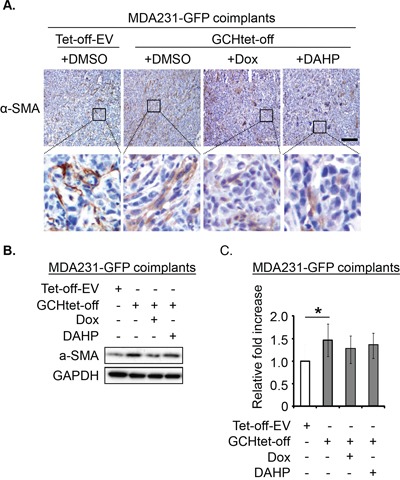
GTPCH potentiates α-SMA expression in breast cancer mouse xenografts Xenografts were done and treated as for Figure 5. Tumor tissue sections were stained immunohistochemically for α-SMA. Scale bar = 100 μM **(A).** Tissue lysates were prepared for SDS-PAGE and immunoblotted with antibodies to α-SMA and GAPDH **(B).** Bands of intensity of α-SMA signal were quantified and normalized with GAPDH. Data are shown as the mean of 5 animals per group ± SEM (*p <0.05 vs. DMSO control tumors) **(C).**

Considering the role of Ang-1 in vessel normalization [22], we analyzed expression of perivascular mural cells attached to tumor vessels. Colocalization of pericyte-associated α-SMA and CD31^+^ endothelial signals was therefore assessed. α-SMA expression (yellow) and CD31 (red) positive vessels overlap (Figure 7E) was approximately 2.5-fold greater in the GTPCH-expressing coimplants than any other groups (Figure 7F). Analysis of them with IHC staining and immunoblotting consistently showed an upregulation of α-SMA in contrast to the weak signal detected in the controls (Figure 8). α-SMA appeared to be diffusely expressed in the stromal fibroblast-like cells (Figure 8A).

Densitometry demonstrated significantly increased α-SMA expression in tumor GTPCH-expressing fibroblast coimplants (approximately 1.5-fold > the control) (Figure 8B-C). Although not significantly different, tumor suppression of GTPCH by Dox or DAHP generated a downward trend in α-SMA expression, suggesting that the GTPCH/BH4 pathway potentiated myofibroblast differentiation *in vivo* but involving other complex extracellular factor participation.

Taken together, our study establishes the protumor paracrine effect of GTPCH expression in fibroblasts in the tumor microenvironment. It facilitates tumor growth, angiogenesis and myofibroblast differentiation *in vivo*.

## DISCUSSION

In this study, we have demonstrated that GTPCH expression is particularly high in ER negative breast tumors in both stroma and epithelium and is a significant predictor of poor outcome in patients. We show that high GTPCH expression in stromal fibroblasts supports breast cancer growth *in vitro* and *in vivo*. The underpinning mechanism involves RTK Tie2 signal transduction on tumor cells mediated by Ang-1 secreted as a result of a GTPCH/Ang-1 interaction in stromal fibroblasts - a previously unrecognized mechanism involved in tumor growth.

Essentially, GTPCH-expressing fibroblasts facilitate breast cancer cell proliferation and migration via Ras signaling and downstream PI3K and MAPK – a similar mechanism that was previously described in endothelial cells when they were incubated by sepiapterin, a precursor of BH4 synthesis [10].

Our pilot study identified the most prominent of effect of CM from GTPCH-expressing fibroblasts on Tie2 phosphorylation in breast cancer cell lines.

RTK Tie2 is normally expressed in endothelial cells and regulates their survival, proliferation and migration by stimulating the PI3K and MAPK pathways [34, 35], the critical signaling that controls angiogenesis initiation [12]. Tie2 expression is also detected in certain types of tumors [14, 17, 36], including breast cancer [37]. We now report a potent extracellular pathway mediated by GTPCH expression in stromal fibroblasts effectively modulating the PI3K and MAPK in breast cancer cells. Our evaluation involved knocking down the endogenous GTPCH gene in human breast fibroblasts and demonstrated significantly decreased breast cancer cell proliferation with CM compared to controls. This observation was substantiated by Tie2 knock-down with siRNA or Tie2 epitope blockage with an Fc fragment antibody to Tie2 receptor. Our data effectively expose a strong connection between GTPCH and Tie2 induction. But we could not rule out Tie2-independent PI3K/MAPK activation by other cytokine stimulation produced by the fibroblasts [1], but primarily initiated by GTPCH. However, most signaling was blocked by Tie2 siRNA or Fc fragment antibody.

We observed a novel GTPCH and Ang-1 interaction in the fibroblasts and a clear effect of GTPCH on Ang-1 secretion. The mechanism of Ang-1 secretion is poorly understood and a possibility raised here is the GTPCH may have a chaperone-like function, but this will require substantial further molecular analysis. Tumor Tie2 phosphorylation on breast cancer growth is consistent with the protumor invasive effect of Ang-1 on Tie2 positive glioma cells, thereby maintaining the stromal and tumor cell communication [38]. However, whether the Ang-1/integrin signaling contributes to tumor PI3K/MAPK activation requires further investigation since both Ang-1 and Ang-2 cell signaling activation can be integrin subfamily-mediated [39, 40].

Our data on coimplantation, during which the stromal fibroblasts and breast cancer cells come in contact *in vivo,* recapitulated studies *in vitro*. GTPCH expression and BH4 synthesis in the tumor microenvironment accelerated tumor growth with significantly increased breast cancer cell proliferation and angiogenesis, in conjunction with PI3K/MAPK activation. We observed that switching off GTPCH expression by Dox or inhibiting GTPCH enzymatic activity by DAHP reduced tumor volumes, Ki67 positive cells, and CD31^+^ microvessel densities in the tumor tissues.

Aberrant Ang-1 overexpression is associated with a large vascular lumen and highly branched vessels [26]. This leads to increased tumor perfusion *in vivo* [41] and to a corresponding increase in mammary tumor growth [26]. Our present study showed that GTPCH-associated Ang-1 expression in tumors with concomitantly increased expression of CD31 and α-SMA. An overlapping signal between CD31 and α-SMA, along with enlarged vessel lumens in the tumor, suggested a role for GTPCH expression in normalizing vessels in breast cancer development *in vivo*.

The present study also described an intriguing observation that the GTPCH/BH4 pathway is associated with α-SMA induction *in vivo*, with its expression diffusely distributed beyond CD31^+^ vessels in the tumor, localized in the fibroblast-like cells, most likely representing a marker of myofibroblast differentiation. Substantial upregulation of α-SMA in the GTPCH-expressing tumors may provide an advantage to a more invasive phenotype because of increased contractility of CAFs linked to α-SMA expression [42].

In conclusion, we have demonstrated a significant correlation between high GTPCH expression and poorer prognosis in breast cancer patients, thus consolidating the clinical significance of the present study. While most previous studies have focused on the angiogenic Tie2 response of endothelial cells to Ang-1 in the tumor microenvironment, our present study shows that the response of tumor Tie2 to a stromal fibroblast-related GTPCH/Ang-1 interaction exhibits a more aggressive phenotype, primarily initiated by GTPCH expression. Furthermore, our investigation using mouse xenografts provides compelling evidence that GTPCH expression favors tumor growth. Our *in vitro* and *in vivo* findings imply that attenuating GTPCH in combination with other agents targeting signaling may act synergistically to block tumor growth.

## MATERIALS AND METHODS

### Cell cultures

NIH3T3 Tet-off-EV and GCHtet-off cells were constructed as described previously [10]. HUVEC cells were grown in our laboratory. MDA-MB231 and BT474 breast cancer cells were purchased from ATCC and chosen for this study because of their low endogenous BH4 with intact RTKs - particularly Tie2 [18]. We modified MDA-MB231 cells to express GFP by lentivirus-mediated gene transfer and established them following expansion. LS110 cells were a normal human breast fibroblast cell line, kindly provided by Professor Valerie Speirs. Before proceeding assays, all cell lines were cultured in serum or growth factor free medium.

### Cell proliferation

GCHtet-off or Tet-off-EV controls were seeded at 1 × 10^5^ cells in a 0.1% BSA pre-coated cell insert (0.4 μm pore size; BD Biosciences) and cultured overnight in DMEM with 10% FBS respectively. The next day, MDA231-GFP were plated at 2 × 10^5^ cells per well in serum-free DMEM ± DMSO vehicle control, Dox (1 μg/ml) or DAHP (5 mM) for a further 48 hours. For BT474, the cells were plated at 1 × 10^5^ per well in RPMI with 10% FBS and incubated with GCHtet-off or Tet-off-EV medium for 72 hours. 20 x phase-contrast time lapse-images were acquired with Incucyte and analyzed (Essen Instrument). They were harvested and counted with a Coulter counter (Beckman).

### Transwell migration

MDA231-GFP was grown in the pre-coated insert (8 μm pore size; BD Biosciences). Cells were plated and treated as above. MDA231-GFP that migrated overnight through the pores to the lower membrane were imaged and 5 random fields taken and quantified with Image J software.

### Western blotting

Immunoblotting was performed using monoclonal antibodies (Cell Signaling) to pAkt (Ser473), Akt, pERK (Thr202/204), ERK, pTie2 (Tyr992), Tie2 and GAPDH. GTP-bound Ras protein were detected as described previously [10]. Screening of 13 RTK families of 42 receptors of human RTK antibody arrays was performed according to the manufacturer's instructions (R&D Systems). For immunoprecipitation, Protein G or A agarose beads (Life Technologies) were bound to antibodies of N or C- terminal Ang-1 (Santa Cruz Biotechnology), or human GTPCH (Sigma), Tie2 (Cell Signaling). Immunostaining for Ang-1 (Acris) and high-affinity anti-rat HA (Roch) was performed. Bands were quantified using Image J software when necessary.

### siRNA knockdown

*GCHsiRNA* was purchased from Dharmacon. The target sequences are as follows: UGGUUUACAUGUCGACUAA, UGGUUUACAUG UUGUGUGA, UGGUUUACAUGUUUUCUGA, UGGU UUACAUGUUUUCCUA. *Tie2siRNAs* and *Ang-1siRNA* were purchased from Santa Cruz Biotechnology, Inc. and designed to specifically knock down the gene expression, and with scramble (SCR) siRNA as the control. We used Lipotectamine RNAiMAX (Life Technologies) for the transfection.

### RNA isolation and qPCR for Ang-1

Total RNA was isolated from cell lysates according to the manufacturer's protocol (Zymo Research). The expression of Ang-1 along with Hprt internal control was assayed by qPCR using Sybr Green (Bioline Reagents Ltd, UK). Primer sequences are as follows: Ang-1_F GGGGGAGGTTGGACAGTAAT, Ang-1_R CGAACCACCAACCTCCTGTT, Hprt_F GCT GGTGAAAAGGACCTCT, Hprt_R CACAGGACTAGA ACACCTGC.

### Cell based ELISA assay for phospho-Tie (Y992)

The experiment was done according to R&D Systems’ instruction. Duplicate readings for each sample were averaged and determined by normalizing fluorescence of pTie2 protein to total Tie2 after subtracting the background.

### Determination of biopterins

In cell and tissue lysates was analyzed by acid-base oxidation followed by fluorometric detection as described previously [10].

### Xenografts

For coimplantation of human breast cancer cells with fibroblasts, 1 × 10^6^ MDA231-GFP ± 2 × 10^5^ GCHtet-off or Tet-off-EV control cells in 100 μl were suspended with an equal volume of Matrigel (BD Bioscience) and were injected subcutaneously into flanks of 6-8 week old female Balb/c SCID mice (Harlan Sprague-Dawley, Inc.) (n = 5) as described previously [10]. All mouse studies were conducted in accordance with protocols approved by the UK Home Office.

### Immunohistochemistry

Formalin fixed paraffin-embedded (FFPE) tissue microarray sections were stained for human GTPCH and scored based on the intensity and proportion of tumor or normal fibroblasts, epithelia, endothelial and inflammatory cells were assessed using a semiquantitative analysis. The intensity of the staining [“no staining” (0), “weak staining” (1), “moderate staining” (2), or “strong staining” (3)] and the percentage of stained cells [0%–10% (1), 10%–50% (2), 51%–80% (3), or 81%–100% (4)] were determined. Intensity was multiplied by percentage to obtain “GTPCH intensity and percentage score” for each tissue sample on TMA as described previously [10].

### Immunofluorescence staining

Cells, grown on glass coverslips, were treated with *Dox* or *DAHP* for 48 hours, or frozen tissue blocks were sectioned at 5 μM. They were fixed, permeabilized and bound to primary antibodies to Ang-1, CD31, α-SMA or HA epitope overnight. Following incubation with secondary antibodies samples were mounted with DAPI and imaged under laser-scanning confocal microscopy (Zeiss ISM510 META).

### Statistics

Results are expressed as mean ± SEM. Statistical significance of differences between means was assessed using Student's unpaired two-tailed *t*-test and Wilcoxon unpaired two-tailored test. A *p* value <0.05 was considered significant. In particular, the computations were performed using *t.test* and *Wilcox.test* R (https://www.r-project.org/) functions. Spearman rank test was used to correlate *GCH1* gene expression with tumor volume and grade. It was also used to correlate GCH1 gene expression with Ang-1. In particular, results have been obtained using *cor.test* R function. Kruskall-Wallis was used to test the association of the gene expression with ER and PR status. Log-rank test and Cox regression were employed to analyze levels of *GCH1*-associated recurrence-free survival; in particular, the *coxph* R function was used. In this analysis, *GCH1* was considered both as binary variable, where patients were divided into 2 groups by median the gene expression, or as continuous variable with Cox analysis, ranked and normalized between 0 and 1.

## SUPPLEMENTARY DATA TABLES AND FIGURES


