# Supplementary material for: Paracrine effect of GTP cyclohydrolase and angiopoietin-1 interaction in stromal fibroblasts on tumor Tie2 activation and breast cancer growth
Source: Oncotarget. 2016 Jan 22;7(8):9353–67. doi: 10.18632/oncotarget.6981 (PMC4891045; doi:10.18632/oncotarget.6981)
Supplement: Supplementary file 1 [file oncotarget-07-9353-s001.pdf]

SUPPLEMENTAL DATA

Supplementary Table S1: Gene array analysis of *GCH1* expression with prognosis of breast cancer in Oxford

Tumor characteristics	Spearman rank test	*GCH1* gene expression
**Tumor size**	Correlation coefficient	0.193
	p value (two-tailed)	**0.017**
	Number of patients	152
**Grade**	Correlation coefficient	0.218
	p value (two-tailed)	**0.013**
	Number of patients	129
**Age**	Correlation coefficient	0.104
	p value (two-tailed)	0.203
	Number of patients	153

Correlation of *GCH1* expression with tumor size and grade in 153 breast cancer cohort in Oxford and a set of published data (Higgins et al., 2010).

REFERENCE

Higgins GS, Harris AL, Prevo R, Helleday T, McKenna WG, Buffa FM. Overexpression of POLQ confers a poor prognosis in early breast cancer patients. Oncotarget 2010; 1: 175-184.

Supplementary Table S2: Multivariate Cox analysis of *GCH1* expression with patient survival in breast cancer

**Correlation of *GCH1* and clinical covariates with recurrence free survival**	***p* value**	**Hazard ratio**
High *GCH1*	**0.024**	3.589
ER positive	0.391	0.664
Menopause	0.894	0.944
Grade	0.638	1.135
Size	**0.010**	1.301
Node	0.801	1.018

Correlation of *GCH1* expression with recurrent free survival in 153 breast cancer patients as described previously (Higgins et al., 2010). Clinical covariates in breast cancer patients in a set of gene array data.

Supplementary Table S3: Oncomine analysis of *GCH1* gene expression in sets of breast cancer cohorts

Dataset name	Dataset size	Analysis of subtype	Comparision (size)	*p* value	t-Test	Fold of changes	Reporter
Bittner	336	Ductal breast carcinoma vs. others	261 vs. 62	**3E-5**	4.2	2.8	204224_s_at
TCGA	593		397 vs. 54	**2E-5**	4.4	2.6	A_24_P167642
TCGA	593	Invasive breast carcinoma vs. normal	76 vs. 61	**5E-12**	7.4	2.9	A_24_P167642
TCGA	593		392 vs. 61	**7E-19**	10.6	3.2	
Wang	286	ER positive vs. negative	209 vs. 77	**4E-06**	-4.7	0.38	204224_s_at
Desmedt	198		102 vs 56	**2E-05**	-4.3	0.33	204224_s_at
Kao	327		204 vs. 123	**4E-12**	-7.2	0.37	204224_s_at
Gluck	158		81 vs. 73	**1E-07**	-5.4	0.36	6502
Hatzi	508	PR positive vs. negative	243 vs 258	**3E-05**	-4.0	0.42	204224_s_at
Kao	327		258 vs. 69	**4E-06**	-4.7	0.39	204224_s_at
Bild	158		101 vs. 57	**2E05**	-4.2	0.39	37944
Ivshina	289		186 vs 61	**5E-06**	-4.7	0.41	204224_s_at
Ivshina	289	Grade I/II/III	68/166/55	**6E-08**			204224_s_at
Yu	96		5/26/63	**9E-05**			204224_s_at
Gluck	158		19/49/69	**6E-07**			6502
Desmedt	198		21/67/70	**2E-07**			204224_s_at
Bittner	336		18/74/126	**2E-06**			204224_s_at
Schmidt	200		29/135/36	**8E-07**			204224_s_at
Hatzis	508		132/180/258	**4E-06**			204224_s_at

Significant results are shown ($p < 0.0001$) for association of *GCH1* expression with histology, biomarker (ER and PR) expression and histological grade (well differentiated/intermediate/poorly differentiated).

Supplementary Table S4: GTPCH expression in murine fibroblasts elevates BH4 levels in breast cancer cells in cultures

Condition	Total biopterin (pmol/mg)	BH4 (pmol/mg)
**MDA231-GFP/Tet-off-EV**	8.8 ± 3.1	4.1 ± 1.7
**MDA231-GFP/GCHtet-off**	62.7 ± 23.2*	18.6 ± 8.7*
**+ DOX**	7.5 ± 2.9	4.0 ± 0.8
**+ DAHP**	8.5 ± 3.6	6.8 ± 0.9

MDA231-GFP was cocultured with GCHtet-off or Tet-off-EV control and incubated with Dox (1 µg/ml), DAHP (5 mM), or DMSO vehicle control for 48 hours. Cell lysates were prepared and biopterin levels were determined by HPLC using both acid-base oxidation with fluorometric detection. Values shown are means of two triplicate determinations ± SEM (*$p < 0.05$ vs. DMSO vehicle control, Dox or DAHP, n = 6).

Supplementary Table S5: GTPCH expression in tumor stromal fibroblasts increases BH4 synthesis in mouse xenografts

Condition	Total biopterin (pmol/mg)	BH4 (pmol/mg)
**MDA231-GFP/Tet-off-EV**	29.1 ± 1.2	11.6 ± 2.1
**MDA231-GFP/GCHtet-off**	91.8 ± 24.1*	24.0 ± 2.0*
**+ DOX**	37.8 ± 1.8	5.5 ± 1.6
**+ DAHP**	40.1 ± 2.9	5.6 ± 1.6

MDA231-GFP cells (1 x 10^6) were coinjected with either GCHtet-off or Tet-off-EV control (2 x 10^5), respectively, and treated with 2 g/L of Dox or DAHP. Tumor tissues were homogenized and biopterin levels were determined by HPLC using both acid-base oxidation with fluorometric detection. Values shown are means ± SEM of 5 animals per group (*$p < 0.05$ vs. Dox or DAHP).

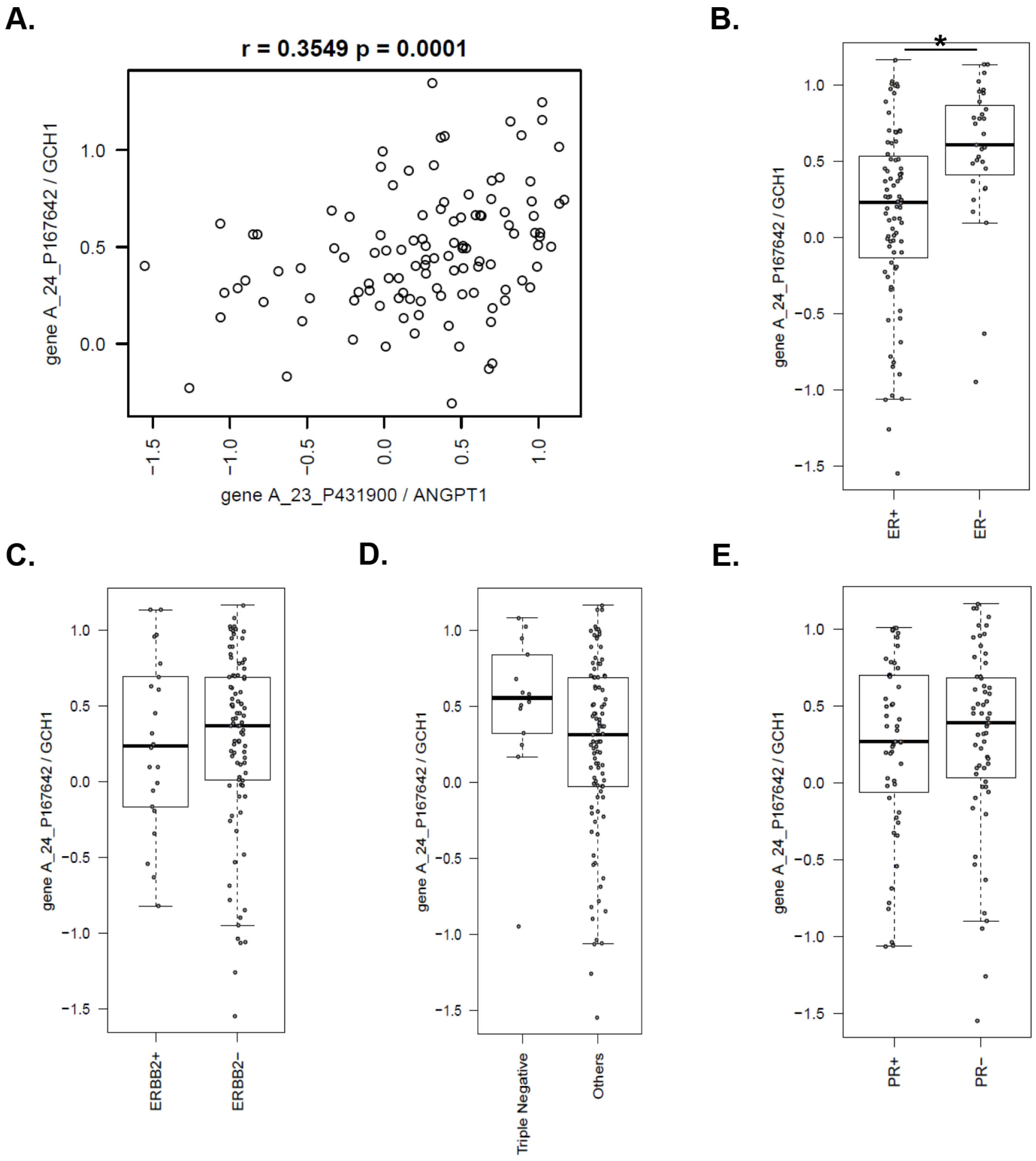

Supplementary Figure S1: GTPCH gene expression in breast tumor stroma and the patient stratification. The *GCH1* gene expression is positively correlated with the Ang-1 **(A).** Spearman's rho=0.3549, *p*-value<0.05. It expresses significantly high in the ER- breast cancer **(B),** but not in any other subtypes of the patients **(C-E).** $p < 0.05$ vs. DMSO control, Dox or DAHP treated tumors.

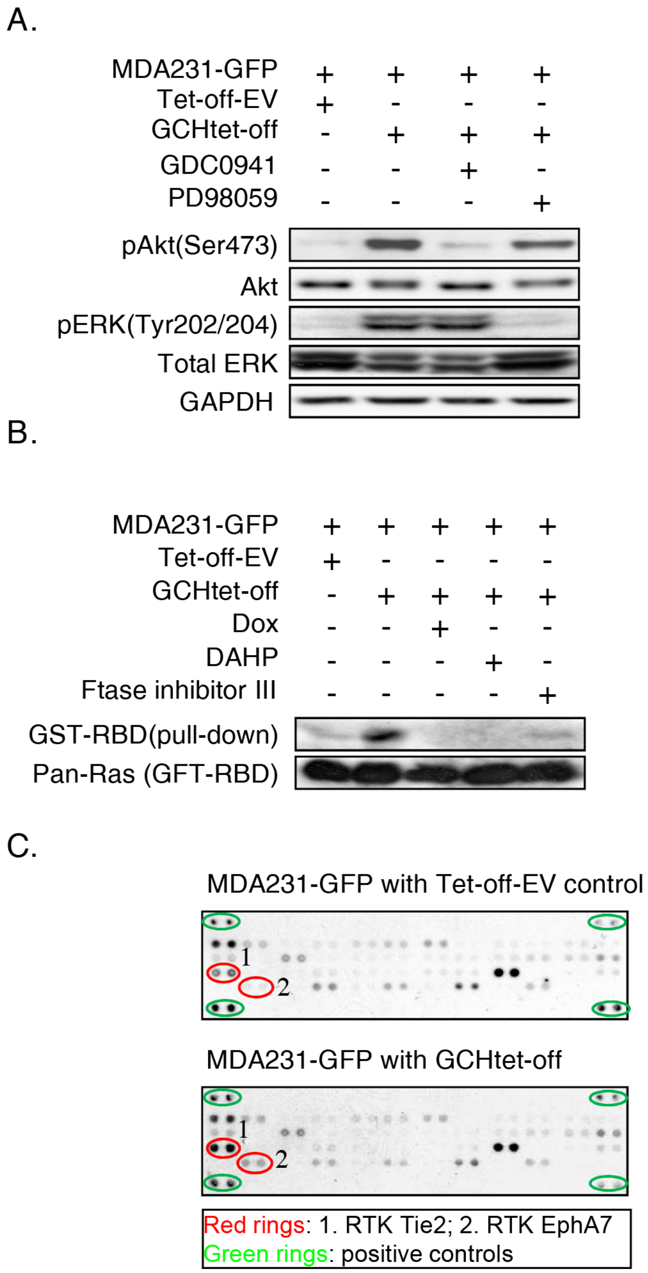

Supplementary Figure S2: GTPCH-expressing murine fibroblasts induce tumor Akt and ERK phosphorylation in conjunction with oncogenic Ras activation. As shown in Figure 2-A, cocultures were either pretreated for one hour with GDC0941 (2 μM), PD98059 (15 μM), the control (DMSO) **(A).** or incubated 48 hours with Dox (1μg/ml), DAHP (5 mM) or Ftase inhibitor III (15 μM) **(B).** MDA231-GFP lysates were prepared for SDS-PAGE and immunoblotted with antibodies to p-Akt (Ser473), Akt, p-ERK (Tyr202/204), ERK, pan-Ras, and GAPDH, respectively (representative 3 independent experiments) (A and B). For human phospho-RTK assay, the lysates were incubated on RTK antibody arrays and immunoblotted with RTK phospho-tyrosine-HRP. Each RTK antibody is spotted in duplicate. Dots in green circles indicate positive controls; the corresponding RTK in red circles is also listed **(C).**

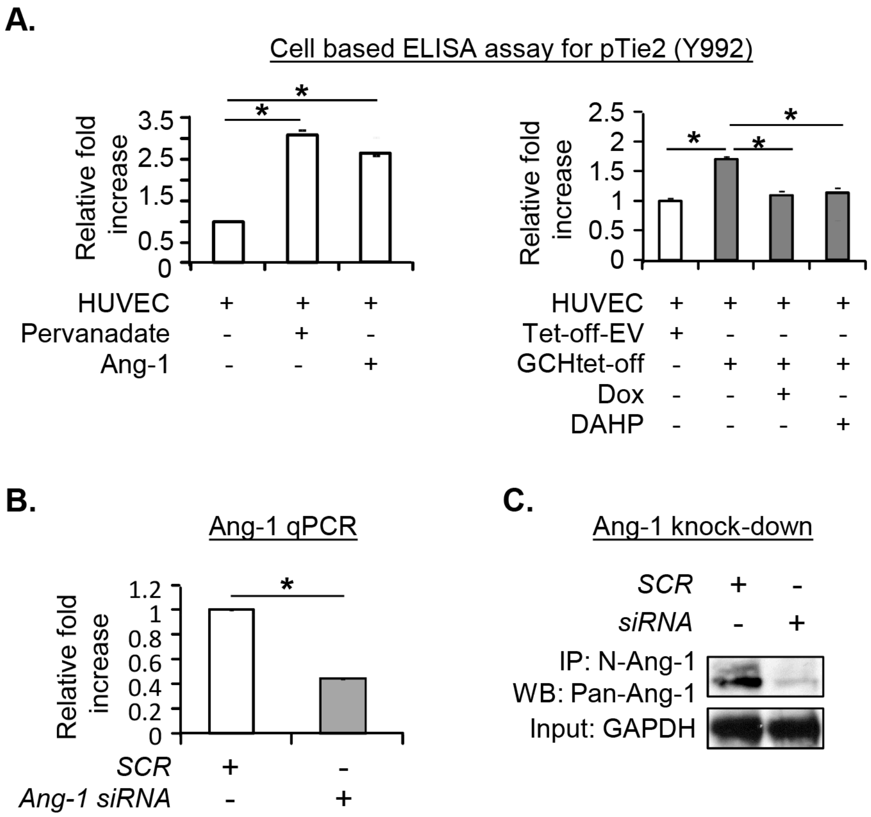

Supplementary Figure S3: Biological effect of GTPCH-induced Ang-1 secretion on tumour Tie2 phosphorylation. (A). 1 x 10^4 of HUVEC cells/well was seeded in a 96-well plate. After incubation with positive controls of pervanadate (100 μM) or recombinant Ang-1 (500 ng/ml), the GCHtet-off or Tet-off-EV media ± the DMSO vehicle control, Dox (1μg/ml), DAHP (5 mM), pTie2 was quantified using cell based ELISA assay. **(B).** GCHtet-off fibroblasts were transfected with *Ang-1siRNA* (10 nM) or *SCRsiRNA* for 72 hours and Ang-1 mRNA were quantified. **(C).** Cell lysates were prepared for immunoprecipitation of Ang-1. They were immunoblotted with antibody to Ang-1 or GAPDH. All data are shown as mean ± SEM (**p* <0.05 vs. the Tet-off-EV control, Dox or DAHP, or *siRNA* knockdown vs *SCRsiRNA* n = 3).

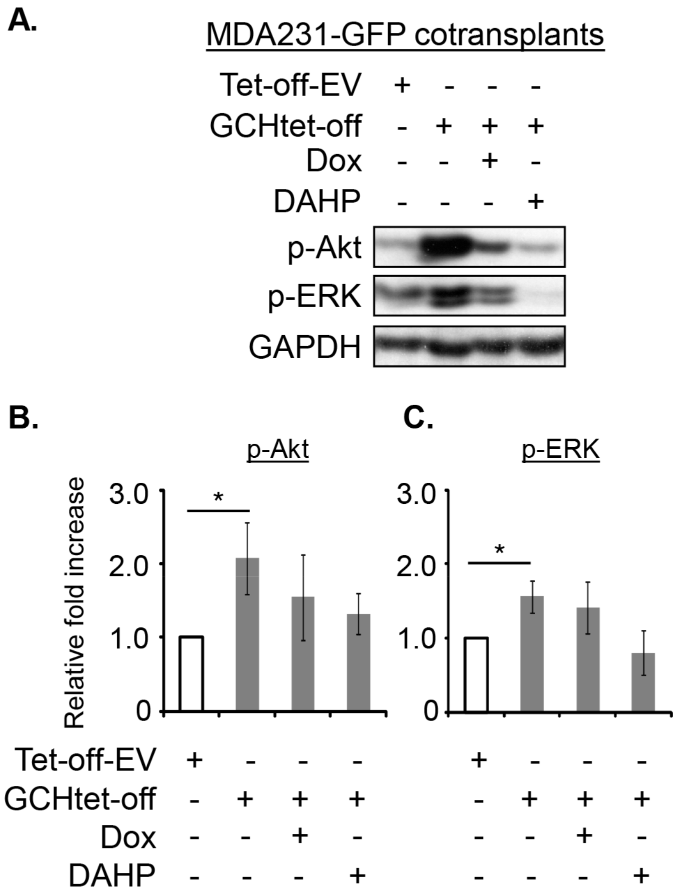

Supplementary Figure S4: Murine fibroblasts expressing GTPCH stimulates tumor Akt/ERK phosphorylation in mouse xenografts. Xenografts were done and treated as for Figure 5. Tumor tissue lysates were prepared for SDS-PAGE and immunoblotted with antibodies to phospho-Akt, phospho-ERK, and GAPDH **(A).** Bands of intensity of the phospho-Akt and phospho-ERK were quantified on ImageJ software and normalized to GAPDH **(B** and **C).** Data are shown as the mean of 5 animals per group ± SEM (**p* <0.05 vs. DMSO control, Dox or DAHP treated tumors).
